# In vitro analysis suggests that SARS-CoV-2 infection differentially modulates cancer-like phenotypes and cytokine expression in colorectal and prostate cancer cells

**DOI:** 10.1038/s41598-024-75718-1

**Published:** 2024-10-19

**Authors:** Alberta Serwaa, Fatima Oyawoye, Irene Amoakoh Owusu, Daniel Dosoo, Aaron Adom Manu, Augustine Kojo Sobo, Kwadwo Fosu, Charles Ochieng Olwal, Peter Kojo Quashie, Anastasia Rosebud Aikins

**Affiliations:** 1 West African Centre for Cell Biology of Infectious Pathogens (WACCBIP), College of Basic and Applied Sciences, University of Ghana, Accra, Ghana; 2 Biochemistry, Cell, and Molecular Biology, College of Basic and Applied Sciences, University of Ghana, Accra, Ghana; 3 Francis Crick Institute, 1 Midland Road, London, NW1 1AT UK

**Keywords:** SARS-CoV-2, Cancer, COVID-19, Spike pseudovirus, Cytokine expression, SARS-CoV-2, Viral host response, Cancer epidemiology

## Abstract

The coronavirus disease 2019 (COVID-19) reportedly exacerbates cancer outcomes. However, how COVID-19 influences cancer prognosis and development remains poorly understood. Here, we investigated the effect of Severe Acute Respiratory Syndrome Corona Virus 2 (SARS-CoV-2), the etiological agent of COVID-19, on cellular cancer phenotypes the expression of cancer-related markers, and various proinflammatory cytokines. We infected prostate (22RV1) and colorectal (DLD-1) cancer cell lines, which express angiotensin-converting enzyme 2 (ACE2), with spike pseudovirus (sPV) and laboratory stocks of live SARS-CoV-2 viruses. After infection, we quantified changes in the cellular cancer phenotypes, the gene expression levels of some cancer markers, including Ki-67, BCL-2, VIM, MMP9*,* and VEGF*,* and proinflammatory cytokines. Phenotypic analysis was performed using MTT and wound healing assays, whereas gene expression analysis was carried out using real-time quantitative PCR (RT-qPCR). We show that SARS-CoV-2 infection impacts several key cellular pathways involved in cell growth, apoptosis, and migration, in prostate and colorectal cancer cells. Our results suggest that SARS-CoV-2 infection does influence various cancer cellular phenotypes and expression of molecular cancer markers and proinflammatory cytokines, albeit in a cell-type-specific manner. Our findings hint at the need for further studies and could have implications for evaluating the impact of other viruses on cancer progression.

## Introduction

Cancer is among the leading causes of death worldwide^1^. While cancer development is multifaceted^2,3^, the complex mechanisms underlying the development of various cancers remain unresolved. Infectious agents, particularly viruses, have been associated with the development of various cancers. Currently, the seven viruses (oncogenic viruses) that have been associated with various cancers include Human papillomavirus (HPV), Epstein-Barr virus (EBV), Kaposi’s sarcoma-associated herpesvirus (KSHV), Human T-lymphotropic virus 1 (HTLV-1), Hepatitis B virus, Hepatitis C virus and Merkel cell polyomavirus^4^. One key mechanism by which oncogenic viruses cause cancer is by inducing the production of immunosuppressive cytokines in cells^5^ and remodeling the host molecular landscapes, enabling viruses to escape immune clearance, and causing uncontrolled cell proliferation. In 2019, the Coronavirus disease 2019 (COVID-19), caused by Severe Acute Respiratory Syndrome Corona Virus 2 (SARS-CoV-2), emerged and infected millions of individuals across the globe. SARS-CoV-2 can infect epithelial and endothelial cells of multiple organs including the lungs, kidneys, intestines, colon, brain, trachea, pancreas, testes, prostate, and blood vessels^6,7^.

During infection, SARS-CoV-2 interacts and binds to host cells that express Angiotensin-converting Enzyme 2 (ACE2), the main entry receptor^8^. The observed presence of ACE2 on many cancer cells^9–12^ has stimulated studies on the role of ACE2 in cancer development^13,14^. However, these studies have yielded contradicting outcomes. On the one hand, some of the studies have shown that *ACE2* expression on cancer cells reduces some cancer-like properties, like proliferation, in breast, colon, lung, non-small cell lung, and pancreatic cancers^15,16^. On the other hand, other studies have suggested that *ACE2* expression enhances the migratory and invasive capabilities of renal adenocarcinomas^17,18^.

The fact that SARS-CoV-2 mainly enters cells through ACE2 receptor raises several questions, including whether the SARS-CoV-2 virus can interact with ACE2 on various cancer cells, and if so, what possible pathways may be dysregulated and how this interaction impacts the progression of the cancer. Although these questions have not been thoroughly investigated, a study suggested that ACE2 interaction with the SARS-CoV-2 spike could have a deleterious effect on the renin-angiotensin system due to changes in its pathway^19^. In cancer, it is predicted that a possible interaction and successful entry could downregulate ACE2 and consequently cause an imbalance as well as elevated bradykinin levels. Either way, downstream processes may lead to reduced anti-proliferation action, angiogenesis, P13K augmentation, and activation of the mitogen-activated protein kinase (MAPK) pathway: all of which have been previously implicated in cancer progression^20^.

Since SARS-CoV-2 may infect *ACE2*-expressing cancer cells and modulate the expression of *ACE2*^21^, we hypothesized that SARS-CoV-2 entry into the cell and its subsequent activities could contribute to cancer development by directly modulating the molecular and immunological aspects of cancer cells. This hypothesis was partly informed by the previous observation that SARS-CoV-2 infection is associated with heightened cytokine production (i.e., cytokine storm)^22^, which is largely a function of chronic inflammation and could influence carcinogenesis^23^.

To test this hypothesis, we harnessed a combination of pseudoviral^24^ and live SARS-CoV-2 assays to investigate how SARS-CoV-2 infection affects (i) cellular cancer phenotypes (ii) the expression of cancer-related markers, and (iii) the expression of various proinflammatory cytokines using various cancer cell lines. Our study shows that SARS-CoV-2 can directly affect cancer cells thereby affecting cancer outcome. These findings together with more mechanistic follow up studies will lead to a better understanding of how SARS-CoV-2 interacts with cancer cells could improve the management of cancer patients who contract COVID-19.

## Results

### ACE2 expression and SARS-CoV-2 infectivity vary across cancer cell types

**Fig. 1:**
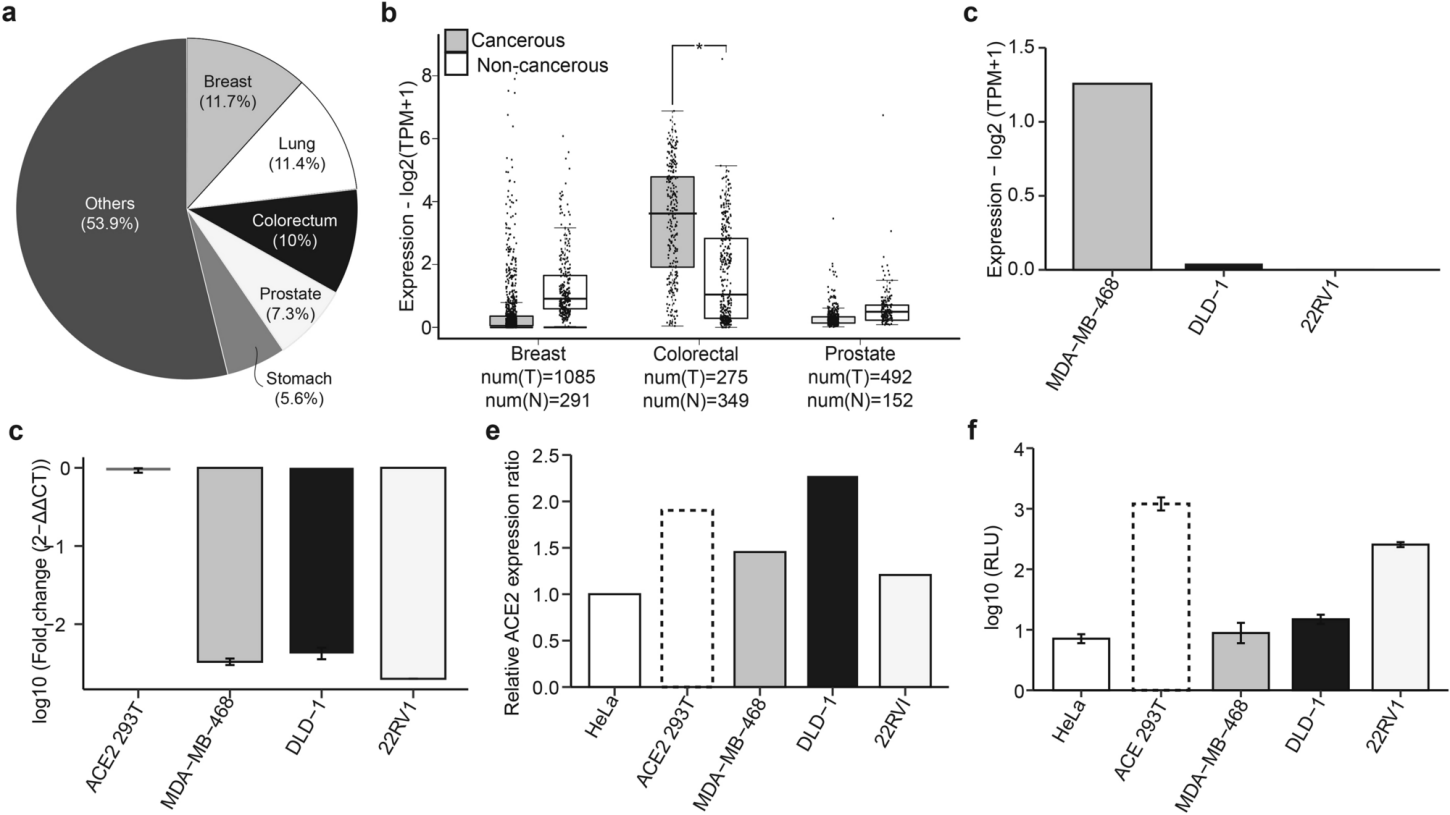
ACE2 expression and SARS-CoV-2 infection of various cancer cells. (**a**) Pie chart showing the proportion of various cancers globally. Data from GLOBOCAN (https://www.uicc.org/news/globocan-2020-new-global-cancer-data). (**b**) Box plots comparing the expression of ACE2 expression in tumor (grey) and corresponding normal (white) breast, colorectal, and prostate tissues. The expression cut-off was set at 1 from the log_2_ fold change (Log_2_FC) with a *p*-value cut of 0.01. Data are presented as a fold change of log expression of ACE2 transcripts per million (Log_2_TPM). The numbers of T = tumor and N = normal tissues are provided. The horizontal line across the box is the median expression while the whiskers depict a 1.5 × interquartile range. (**c**) Bar plot showing ACE2 expression in MDA-MB-468, DLD-1, and 22RV1 cell lines. The values were transformed to log2(TPM + 1). Data from DEPMAP (https://depmap.org/portal/interactive/). (**d**) Bar plots summarizing the mRNA expression of ACE2 transcripts across the three cell lines were determined using RT-qPCR. The data represent log-transformed fold changes for at least three independent experiments. Error bars depict the standard error of the mean (SEM). (**e**) Bar plot showing expression of ACE2 protein in the three cell lines measured using dot blot assay. Dot intensities were quantified with ImageJ/Fiji and expressed as the relative protein expression ratios of net band to net loading control. (**f**) Bar plots showing the infectivity of various cell lines with SARS-CoV-2 spike pseudovirus (sPV). The infectivity measurements were performed using luciferase assay on a GloMax luminescence plate reader. Infectivity was expressed as log10 relative light unit (RLU). Errors bars represent SEM. ACE2 293 T, a cell line stably expressing ACE2, and HeLa cells, known to lack ACE2, were used as controls.

First, we set out to select cancer cell lines for our experimental analyses. Using a combination of in silico and in vitro analyses, we assessed the expression of *ACE2* on several cancer cell lines and the relative permissibility of the cells to infection by SARS-CoV-2. For in silico analyses we used two approaches: firstly, data from the Gene Expression Profiling Interactive Analysis 2 (GEPIA2; http://gepia2.cancer-pku.cn/#analysis) software was used to compare the expression of ACE2 between cancerous and non-cancerous breast, colorectal, and prostate tissues, the three leading cancers across all ages and genders worldwide (Fig. 1A; https://www.uicc.org/news/globocan-2020-new-global-cancer-data), and originate from cells that may be susceptible to SARS-CoV-2^25^ aside from lung cells. Our GEPIA2 analysis revealed that *ACE2* expression was significantly higher in cancerous colorectal tissues compared to normal tissues (Fig. 1B). However, there was no difference in *ACE2* expression between cancerous prostate or breast tissues relative to their respective normal tissues (Fig. 1B). We next extracted and analyzed available data from DEPMAP (https://depmap.org/portal/interactive/), a database that contains cancer dependencies, for three cell lines: MDA-MB-468, DLD-1, and 22RV1, representing breast, colorectal, and prostate cancers, respectively. The analysis confirmed the expression of *ACE2* in these three cell lines (Fig. 1C). Subsequently, *ACE2* expression in these three cell lines was further confirmed in the lab, using real-time quantitative PCR (RT-qPCR; Fig. 1D) and dot blotting (Fig. 1E and Fig. S1). Altogether, these analyses revealed that the three cell lines (MDA-MB-468, DLD-1, and 22RV1) expressed *ACE2,* albeit at different relative levels. Furthermore, the susceptibility of these cell lines to SARS-CoV-2 was investigated. To this end, we performed in vitro infection of the three cell lines with luciferase-labeled SARS-CoV-2 spike pseudovirus (sPV). We observed a significant infection in the prostate (22RV1) and colorectal (DLD-1) cells but not in the breast cancer (MDA-MB-468) cell lines (Fig. 1F). Based on these results, 22RV1 and DLD-1 cell lines were selected for subsequent experiments.

### SARS-COV-2 infection modulates the proliferation, viability, and migration of prostate and colorectal cancer cells

**Fig. 2:**
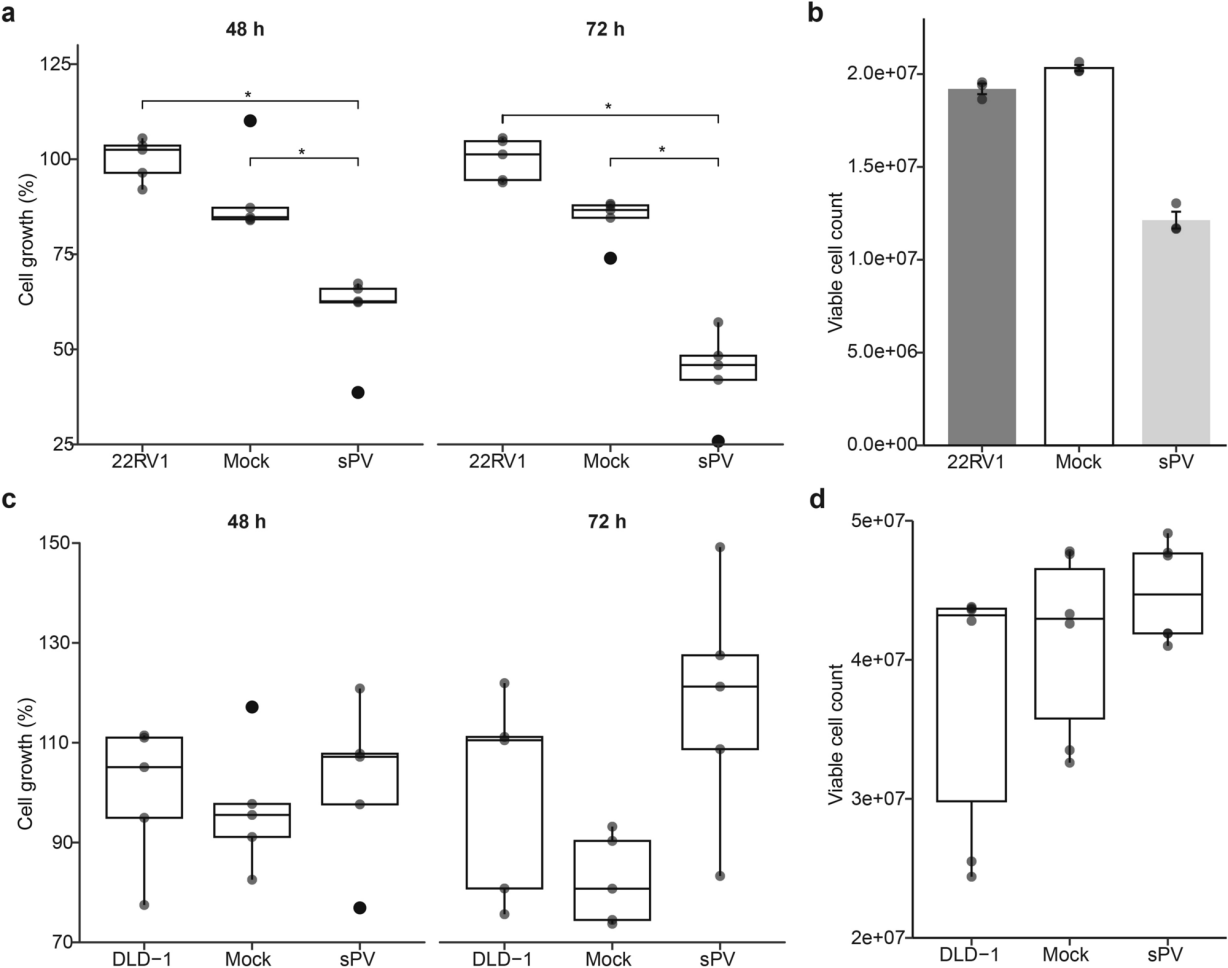
Effect of SARS-CoV-2 spike pseudovirus (sPV) infection on the proliferation and viability of prostate and colorectal cancer cell lines. (**a**) Box plots showing the proliferation of 22RV1, a prostate cancer cell line, infected with mock and sPV for 48 and 72 h. Data are expressed as the percentage cell growth calculated by (mean absorbance of test/mean absorbance of control) × 100. Box plots represent the median and upper and lower quartiles of the distribution whereas whiskers represent 1.5 times the interquartile range. (**b**) Bar plots showing the viability of 22RV1, a prostate cancer cell line, infected with mock and sPV for 72 h. The data is a representation of the Mean ± SEM of experimental groups. (**c**) Box plots showing the proliferation and viability of DLD-1, a colorectal cancer cell line, infected with mock and sPV for 48 and 72 h. Data are expressed as the percentage cell growth calculated by (mean absorbance of test/mean absorbance of control) × 100. (**d**) Box plots showing the viability of DLD-1, a colorectal cancer cell line, infected with mock and sPV for 72 h. Box plots represent the median and upper and lower quartiles of the distribution whereas whiskers represent 1.5 times the interquartile range.

To determine the effect of SARS-CoV-2 infection on the proliferation, viability, and migration of prostate (22RV1) and colorectal (DLD-1) cancer cell lines, we harnessed different experimental approaches using SARS-COV-2 sPV and live SARS-CoV-2 virus (LV). First, we infected the 22RV1 and DLD-1 cell lines with sPV and assessed changes in the cell proliferation and viability relative to mock-infected (an empty vector comprising of PCSFLW and p8.91 backbone) and uninfected cells only control. Our analysis revealed that 22RV1 cells infected with sPV for 48 or 72 h exhibited significantly decreased proliferation (Fig. 2A). consequently, we observed a considerable reduction in viable 22RV1 cells after infection with sPV (Fig. 2B). On the other hand, infection with sPV did not significantly affect the proliferation and viability of DLD-1 cells although there is an increasing trend of proliferation and viability after infection (Fig. 2C,D).

**Fig. 3:**
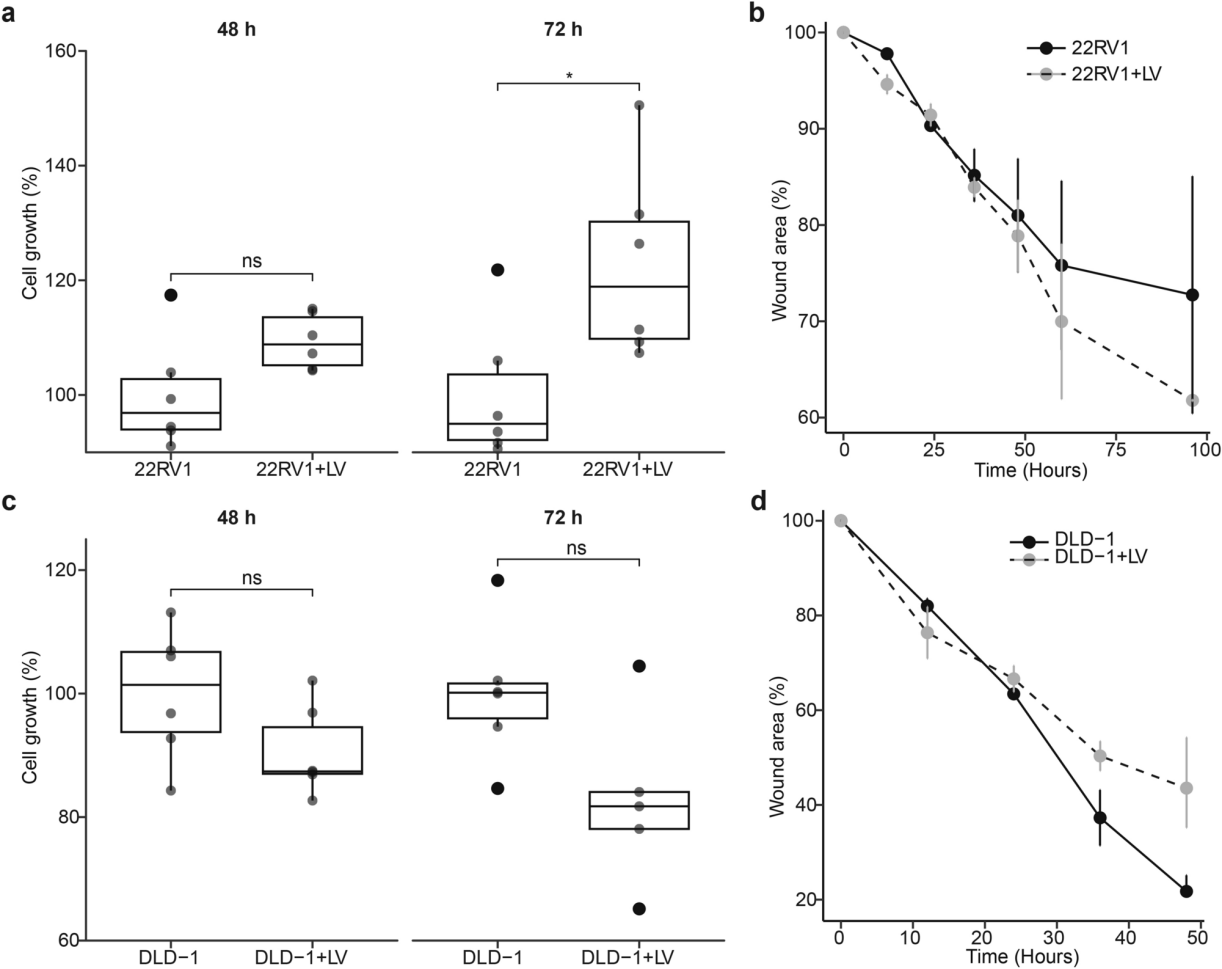
Effect of SARS-CoV-2 infection on prostate and colorectal cancer cell proliferation and migration. (**a**) Box plots showing the proliferation of 22RV1, a prostate cancer cell line infected with LV for 48 and 72 h. Data are expressed as the percentage cell growth calculated by (mean absorbance of test/mean absorbance of control) × 100. Box plots represent the median and upper and lower quartiles of the distribution whereas whiskers represent 1.5 times the interquartile range. (**b**) Line plots showing the changes in the wound area in LV infected and uninfected 22RV1 cells across different time points. Error bars represent the SEM. (**c**) Box plots showing the proliferation of DLD-1, a colorectal cancer cell line infected with LV for 48 and 72 h. Data are expressed as the percentage cell growth calculated by (mean absorbance of test/mean absorbance of control) × 100. Box plots represent the median and upper and lower quartiles of the distribution whereas whiskers represent 1.5 times the interquartile range. (**d**) Line plots showing the changes in the wound area in LV-infected and uninfected DLD-1 cells across different time points. Error bars represent the SEM.

Next, we set out to interrogate the effects of the live SARS-CoV-2 viruses (LV) on the proliferation and migration of 22RV1 and DLD-1 cell lines. Unlike sPV, the live virus infection significantly increased the proliferation of 22RV1 cells relative to the uninfected 22RV1 cells (Fig. 3A). Consistent with this, LV infection resulted in a slight increase in the migration (wound closure) of 22RV1 cells (Figs. 3B and S2A). In contrast to the sPV infection, 48 h or 72 h of LV infection considerably reduced the proliferation of DLD-1 cells compared to the uninfected DLD-1 cells (Fig. 3C). Furthermore, we observed that the wound closure in LV-infected DLD-1 cells was slower than in uninfected cells (Figs. 3D and S2B). Altogether, these findings suggest that the SARS-CoV-2 infection has a different effect on the cancer phenotypes in prostate and colorectal cancer cells, implying that SAR-CoV-2 infection can either promote or decrease cancer development depending on the cell type and whether a whole virus or components of the virus is used.

### SARS-COV-2 LV infection modulates key markers of cell growth and survival in a cell-dependent manner

**Fig. 4:**
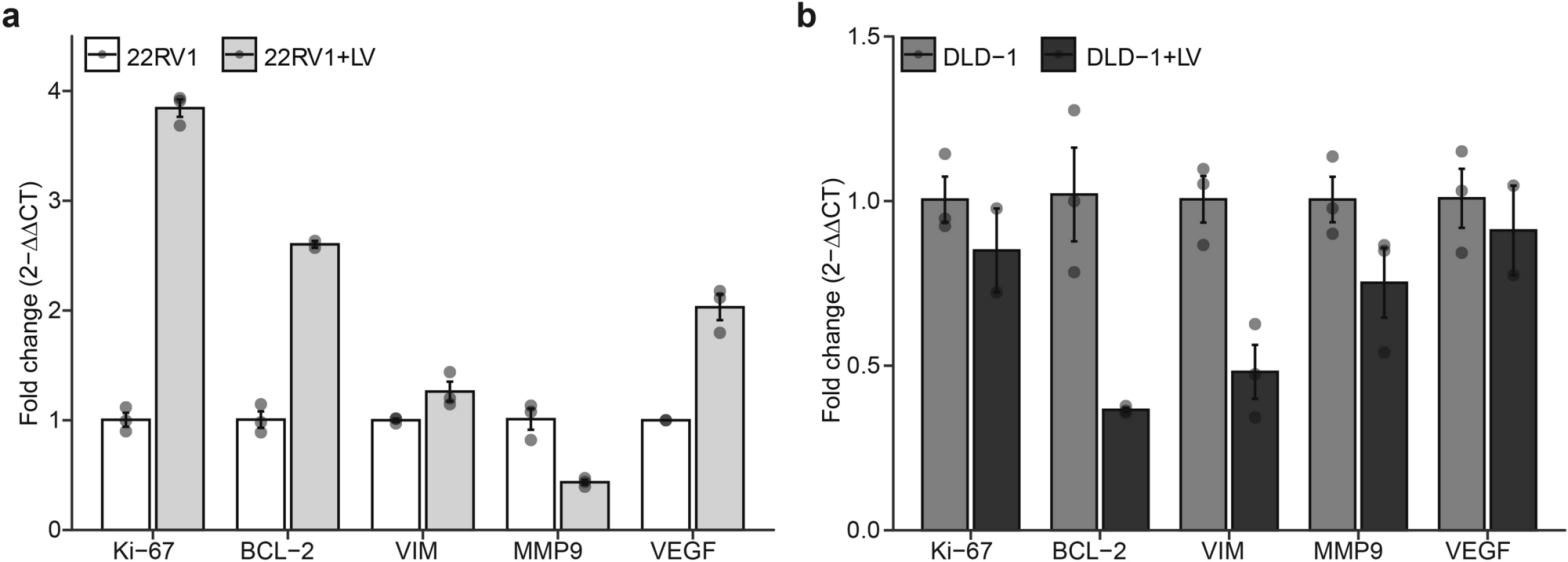
Effect of SARS-CoV-2 infection on the expression of various cancer markers in prostate and colorectal cancer cells. (**a**) Bar plots showing the relative expression of proliferative, apoptotic, migratory, and angiogenic markers in 22RV1 infected with LV. Data are expressed as the fold change calculated by 2^-ΔΔCT^. Error bars depict SEM. (**b**) Bar plots showing the relative expression of proliferative, apoptotic, migratory, and angiogenic markers in DLD-1 infected with LV. Data are expressed as the fold change calculated by 2^-ΔΔCT^. Error bars depict SEM.

To gain insights into possible cancer pathways that may be hijacked during SARS-CoV-2 infection, we evaluated the expression of molecular markers for cell proliferation (i.e., *Ki-67*), apoptosis (i.e., *BCL-2*), migration (i.e., *VIM & MMP9*), and angiogenesis (i.e., *VEGF*) in LV-infected 22VR1 and DLD-1 cells using RT-qPCR assay. Our data showed that infection of 22RV1 with LV increased the expression of Ki-67 *and* BCL-2, relative to the uninfected cells control (Fig. 4A) in line with the observed increase in the proliferation of this cell line previously observed in Fig. 3A. Also, we noticed increased expression *of* VIM and VEGF upon infecting the 22RV1 cells with LV except for MMP9, which was downregulated upon infection (Fig. 4A). Moreover, we observed downregulation of Ki-67, BCL-2, VIM, MMP-9*,* and VEGF expression upon infecting DLD-1 cells with LV (Fig. 4B).

### SARS-CoV-2 increases the expression of certain proinflammatory cytokines in prostate and colorectal cells

**Fig. 5:**
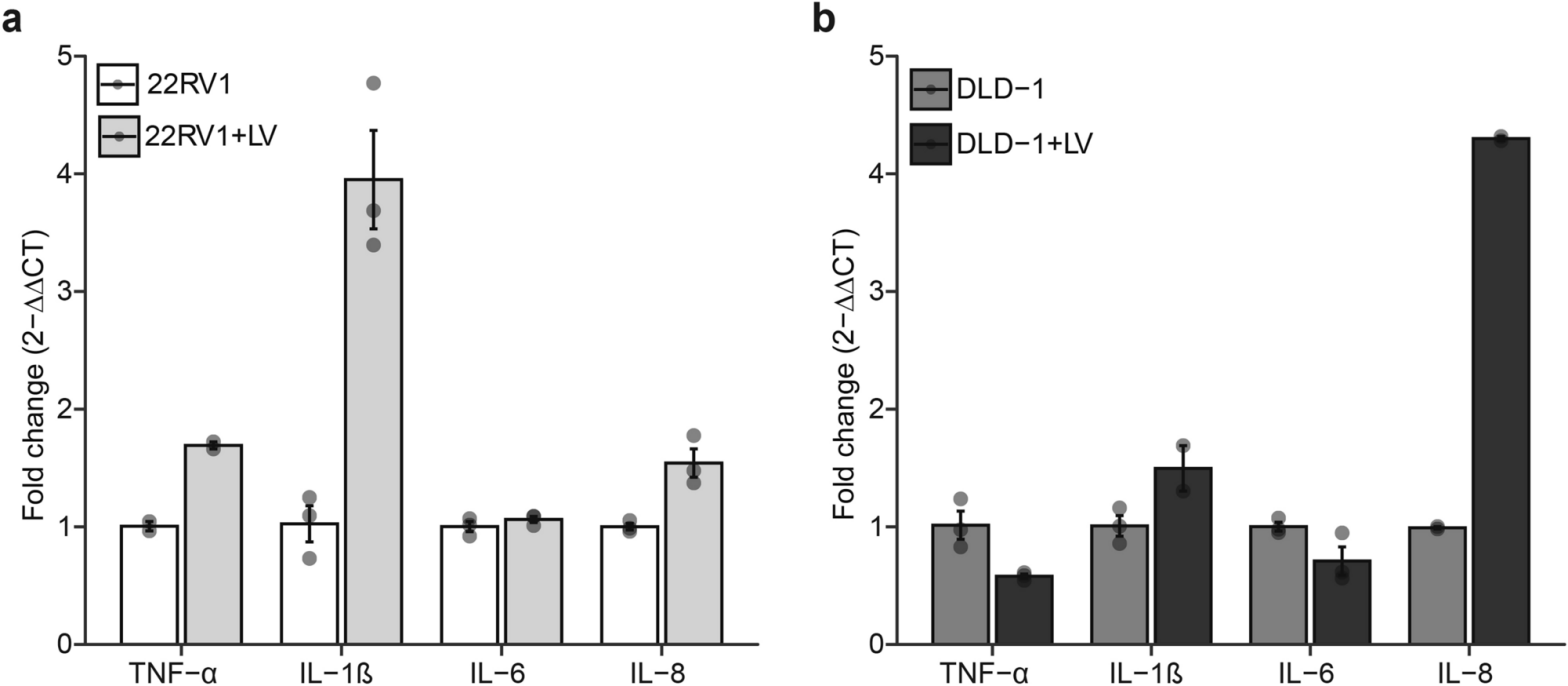
SARS-CoV-2 increases certain proinflammatory cytokine expression. (**a**) Bar plots showing the relative expression of four proinflammatory cytokines in 22RV1 infected with LV. (**b**) Bar plots showing the relative expression of four proinflammatory cytokines in DLD-1 infected with LV. Data are expressed as the fold change calculated by 2^-ΔΔCT^. Error bars depict SEM.

The effect of live SARS-CoV-2 infection on the gene expression of proinflammatory cytokines in the prostate (22RV1) and colorectal (DLD-1) cancer cell lines was determined. The gene expression levels of four cytokines; TNF-α, IL-6, IL-1β, and IL-8 were tested in the cell lines after infection. Our analysis revealed that LV infection considerably increased the expression of TNF-α, IL-β, and IL-8 genes while levels of IL-6 were unchanged in 22RV1 cells relative to the uninfected cells (Fig. 5A). In DLD-1 cells, the expression of IL-1β and IL-8 were upregulated while TNF-α and IL-6 expression were downregulated after LV infection (Fig. 5B). These data suggest that SARS-CoV-2 infection may induce gene expression of some cytokines in cancer cells thereby influencing the immune response and the tumor microenvironment.

## Discussion

SARS-CoV-2 infection has been shown to worsen cancer outcomes^26^. However, the effect of this infection on cancer phenotypes and the immunological profile of cancer cells remains unclear. Here, we set out to investigate the effects of SARS-CoV-2 on cellular cancer phenotypes, expression of cancer markers, and proinflammatory cytokines.

The enzyme *ACE2,* which has been shown to facilitate entry of the SARS-CoV-2 virus into host cells has a wide tissue tropism^27^. In this study, we observed variable expressions of the *ACE2* gene across different cancer tissues and cell lines. This emphasizes the importance of considering tissue-specific factors when investigating the interaction between SARS-CoV-2 and host cells^28^. Despite prostate cancer tissues exhibiting low *ACE2* expression levels, the infectivity of 22RV1, a prostate cancer cell line, was high. This suggests that other host intrinsic factors may influence SARS-CoV-2 entry and infectivity of cancer cells. A variety of factors, such as mutations, co-receptors, cell health and cycle, and viral factors among others may influence viral entry^29^. Therefore, follow-up studies will be needed to uncover other host and viral factors that could be affecting the entry of SARS-CoV-2 into *ACE2*-expressing cancer cells.

Furthermore, our investigation yielded insights into the impact of SARS-CoV-2 on the proliferation and migration of some cancer cell types. We observed that SARS-CoV-2 infection either increases the percent growth, and expression of the mRNAs *Ki-67* and *BCL-2* or decreases it depending on the cell type. *Ki-67* is a common proliferation marker, an important prognostic marker of cell growth, and may reflect the aggressiveness of cancers^30^. The *BCL-2* gene, on the other hand, can be used as both an apoptotic and proliferation marker. This is because cells resisting cell death and actively proliferating highly express the *BCL-2*^31,32^. Our study indicates that infection has a distinct effect on the expression of genes associated with proliferation in these cancer cell lines. The significant reduction in 22RV1 proliferation and viability after sPV infection observed in this study is comparable with a recent study where the SARS-CoV-2 spike inhibited the growth and promoted apoptosis of the LNCaP prostate cell line^33^. While the impact of packaging proteins on pseudoviruses on cancer cell properties remains largely unexplored, it is crucial to utilize live viruses to accurately assess the effects of the SARS-CoV-2 virus on these cell lines. Hence, in a non-invasive prostate adenocarcinoma, such as the 22RV1 cell line, interaction with the live SARS-CoV-2 may contribute to poor prognosis of the disease since LV infections could cause an increase in cell growth and expression of markers associated with proliferation. Additionally, we found that the infection may induce migration or suppress cellular migratory properties and migratory markers (i.e., *VIM* and *MMP9*). *VIM* is known to encode cytoskeletal proteins usually found in mesenchymal tissues. Its higher expression indicates the acquisition of mesenchymal phenotypes through epithelial-mesenchymal transition (EMT), indicative of metastasis. *MMP-9* proteolytically digests the ECM molecules to enable cell migration. Notably, SARS-CoV-2 infection may regulate the migration of the cell lines through the upregulation or downregulation of *VIM* and *MMP9* genes. In this present study, we found that the SARS-CoV-2 infection reduced percent cell growth and its related markers of the DLD-1 cell line. Although not much has been done to investigate the effects of infection on colorectal cell lines, especially the DLD-1 cell line, in a recent study, the virus had no distinct effect on HLA expression levels in Caco2 but affected their levels in Calu3 cell lines^34^. In another study, the virus was found to induce EMT through the secretion of TGF-β1 in some colorectal cancer cell lines using the S and N protein peptide^35^. These findings, although not comparable to our findings, demonstrate that the virus can affect the phenotypic and molecular characteristics of colorectal cancer cells. Nonetheless, our findings suggest that the effects of the SARS-CoV-2 virus on cancer cell behavior are cell-type dependent. Our finding highlights the need to consider the cell-type specific responses when studying the impact of SARS-CoV-2 on cancer cells.

Various types of cytokines are implicated in the COVID-19-related cytokine storm^36^. Here, we analyzed the gene expression of some proinflammatory cytokines including TNF-α, IL-1β, IL-6*,* and IL-8. As expected, SARS-CoV-2 infection generally elevated the expression of some of these cytokines, implying that the viral infection may trigger a cytokine storm-like response, which has been associated with severe COVID-19 cases^37^. Within the tumor microenvironment, cytokines may act as drivers of tumorigenesis or as inhibitors of tumors. For instance, *IL-6* has been involved in signaling that promotes cancer progression and survival^38^ and could activate pathways and proteins implicated in EMT. *IL-1β* has been shown to interact with other cytokines to initiate a cascade of events that results in promoting EMT. TNF-α within the tumor microenvironment can cause tumor proliferation as well as metastasis through EMT activation^39^. Cytokines such as IL-1, IL-6, and IL-8 have been shown to cause an acceleration in the progression of an existing prostate tumor^40^. Therefore, the increase in mRNA levels post 22RV1 infection indicates that SARS-CoV-2 infection may contribute to prostate cancer progression by enhancing the release of some of these cytokines. Our data shows an increase in TNF-α and IL-6 but a decreased expression in IL-1β and IL-8 post DLD-1 infection. TNF expression in colorectal cancers is mostly associated with better prognosis^41^. Hence, the increase in TNF-α expression might be implicated in the decreasing phenotypic effect of LV-infection in DLD-1 cells. IL-6, IL-1β, and IL-8 all of which are known to be implicated in tumor progression were either downregulated or non-significantly upregulated. Our findings suggest that SARS-CoV-2 LV infection may downregulate factors promoting DLD-1 cell growth and upregulate some that might promote regression. However, the overall impact of these cytokines on cancer is complex, given that some factors like *IL-6* and TNF-α can have both pro-tumorigenic and anti-tumorigenic effects. Also, measuring cytokine gene expression and not the protein expression levels; a limitation to the study, may impair the accurate representation of cytokines expressed and the justification of how they are affecting the cell lines. The virus might directly interfere with cellular signaling pathways to modulate gene and cytokine expression. Alternatively, the changes might be secondary effects resulting from the cell’s immune and stress responses to the infection. Continuous and increased cytokine production could also be brought about by *ACE2*-mediated activation of *NF-κB*. This signaling could release *IL-6* and *TNF-α*, IL-8, and other chemokines like *VEGF*^42,43^. This study has however provided the basis for understanding how SARS-CoV-2 may be affecting some phenotypic markers and cytokine gene expression. Nonetheless, further studies may be conducted to employ other methods to measure these variables.

It is worth noting that our study revealed some differences between sPV and LV in terms of proliferation and viability in the same cell type. These differences can be attributed to the ability of live viruses to replicate, spread, and present a complete viral replication cycle thus exposing the host cell to other viral proteins and nucleic acid, compared to pseudovirus, which often undergoes a single round of replication and only expresses the spike protein. These observations suggest that the viral components or the infection dynamics may play a crucial role in determining the cellular responses, and the use of a pseudovirus may not fully capture the effects of the authentic SARS-CoV-2 virus.

Although our study was carefully designed and provides valuable information regarding the potential role of SARS-CoV-2 infection in cancer development, we acknowledge several limitations. First, the study is conducted in vitro, which may not fully reflect the real complex interactions and microenvironmental factors present in the body that may cooperate to enhance cancer development. Secondly, even though we evaluated multiple cell lines (Fig. S1) for inclusion in this study, only two cancer cell lines were used to address our research questions. We acknowledge that two cell lines may not be sufficient to draw broad conclusions about the effects of SARS-CoV-2 on cancer progression. Third, our study has not devolved into the possible underlying molecular mechanisms or signaling pathways through which SARS-CoV-2 infection may modulate the cancer-related processes, limiting the mechanistic understanding of the observed effects. Fourth, the study only evaluated the expression of various markers at the gene level. Additional protein expression and functional analyses could have provided a more comprehensive picture of the molecular effects of SARS-CoV-2 infection on cancer cells. We hope future students will address these limitations.

In summary, our study sheds light on the complex interplay between SARS-CoV-2 and cancer cell behavior. While *ACE2* expression plays a role in viral entry, it is not the sole determinant of infectivity. The observed effects on proliferation and migration vary between different cancer cell lines, suggesting cell-type-specific responses to viral infection. Our molecular analysis highlights potential candidate genes that may mediate these effects, warranting further investigation. Additionally, the proinflammatory cytokine response to infection raises questions about the virus’ influence on the tumor microenvironment. Understanding these interactions is critical for elucidating the impact of SARS-CoV-2 infection on cancer patients and may have implications for therapeutic strategies and patient management in the context of COVID-19 and cancer. Our findings hint at the need for further research to unravel the intricate mechanisms underlying these observations and their clinical relevance. This could ultimately lead to the discovery and design of effective therapeutics targeting the adverse effects of SARS-CoV-2 or COVID-19 in cancer patients.

## Materials and methods

### In silico ACE2 gene expression analysis

Differential expression of the *ACE2* gene was analyzed using Gene Expression Profiling Interactive Analysis 2 (GEPIA2; http://gepia2.cancer-pku.cn/#analysis) software. The program performs gene expression analysis based on 9736 tumors from The Cancer Genome Atlas (TCGA) and 8587 normal samples from The Genotype-Tissue Expression (GTEx) databases, using standard RNA-sequencing data. Briefly, *ACE2* gene expression analysis was carried out utilizing the defined sample selections: Breast Adenocarcinoma (BRCA), Colon adenocarcinoma (COAD), and Prostate adenocarcinoma (PRAD). The expression profiles were presented as box plots comparing the tumor tissues to the normal tissues of the selected samples. Next, we used Dependency Map (DEPMAP) database (https://depmap.org/portal/interactive/) to extract cell line-specific *ACE2* expression profiles for MDA-MB-468, DLD-1, and 22RV1. A cell lines list containing our cell line of interest was made using the cell line selector and analysis was performed based on the Expression Public 23Q4 dataset.

### Cell lines and cell culture

MDA-MB-468, ACE2-293T^*^, and HeLa cells were grown and maintained in Dulbecco’s Modified Eagle Medium (DMEM) while DLD-1 and 22RV1 were grown and maintained in Roswell Park Memorial Institute (RPMI) 1640, all supplemented with 10% fetal bovine serum (FBS) and 1% penicillin–streptomycin (Pen-strep) (all purchased from Gibco-life technologies, Carlsbad, CA, USA) at 37 °C and 5% CO_2_. DLD-1 cells were kindly provided by Professor Regina Appiah-Opong of the Clinical Pathology Department, Noguchi Memorial Institute for Medical Research, Ghana. All the cell lines originated from the American Type Culture Collection (ATCC).

### SARS-CoV-2 spike pseudovirus (sPV) production

SARS-CoV-2 spike pseudovirus was produced using the calcium phosphate transfection method as described by Chen and colleagues^44^. Briefly, confluent HEK 293 T cells were co-transfected with pcDNA 3.1 SARS-CoV-2 Spike (#145032), firefly luciferase-expressing plasmid PCSFLW, and a lentiviral packaging plasmid p8.91 (all from Addgene, USA). The transfected cells were cultured for 24 h at 37 °C with 5% CO_2_ after which media was replenished. Media containing sPV were harvested 48- and 72-h post-transfection and filtered through a 0.45 μm syringe-driven filter. Harvested viruses were stored at − 80 °C until use. Mock PV was made by transfecting HEK 293 T cells with the firefly luciferase-expressing plasmid PCSFLW and a lentiviral packaging plasmid p8.91 without the pcDNA 3.1 SARS-CoV-2 Spike plasmid.

### SARS-CoV-2 pseudovirus luciferase infectivity assay

Briefly, ACE2-293 T cells were seeded and incubated for 24 h at 37 °C with 5% CO_2_. About 300 μL of PV was added to each well and cultured for 48 h at 37 °C with 5% CO_2_. Per the manufacturer’s instructions, the luciferase activity was determined using the One-Step™ Luciferase Assay System (BPS Bioscience, USA; #60690-2). A 200 μL working solution was prepared by making a 1:100 dilution of the luciferase reagent substrate (B) and luciferase reagent buffer (A). The working solution was added to each well and incubated for 15 min while rocking gently. Luminescence was measured using the GloMax®-Multi detection system (Promega, USA). Cells only and cells with the mock virus were used to determine the background signal. Successfully produced PV’s were quantified, as described previously by our team,^24^ to determine their working dilutions at a 2.50 × 10^4^ RLU.

### Live virus infections

Live virus infections were performed for all assays. Briefly, laboratory stocks of live SARS-CoV-2 D614G virus (LV) isolated from respiratory samples of COVID-19 patients were a kind donation from the Global Immunology and Immune Sequencing for Epidemic Response (GIISER)-Ghana project led by Dr. Peter Quashie. For all the live virus assays, cells were infected at a multiplicity of infection (MOI) of 0.02 based on a TCID_50_ titration of the virus stock at the time of viral isolation. All infections were done in BSL-3 laboratory facility following all the good laboratory practices for live virus handling at the Noguchi Memorial Institute for Medical Research, Ghana.

### Gene expression analysis

For all gene expression analysis, total RNA was extracted from cell lines using the Zymo Quick-RNA™ Miniprep Plus Kit (Zymo Research, Irvine, CA, USA; #R1057) following the manufacturer’s protocol. RT-qPCR assay was performed using the Luna Universal One-Step RT-qPCR kit (New England Biolabs, Ipswich, MA, USA; #N0491) with primers specific to the target genes (Table S1). *GAPDH* was used as an internal control. Primers were diluted with 1X TAE buffer to a working concentration of 10 Mm. Reverse transcription and amplification were performed on the QuantStudio™ RT-PCR System (Thermo Fisher Scientific, Carlsbad, CA, USA) using the following cycling conditions: reverse transcription (55 °C for 15 min), initial denaturation (95 °C for 1 min), 40 cycles of denaturation (95 °C for 15 s), annealing (different temperatures (see Table S1) depending on target gene for 15 s), and extension (60 °C for 1 min). QuantStudio™ Design & Analysis Software (Life Technologies, Carlsbad, CA, USA) was used to obtain cycle threshold (CT) values. The expression levels of genes were calculated by subtracting the average CT of *GAPDH* from the average CT of target genes. The relative expression levels of each gene were determined using the 2^-ΔΔCT^ method. The mRNA level for the control was expressed as 1.0, and all other quantities were expressed as fold differences relative to the control.

### Dot blot assay of *ACE2* protein expression

To complement the *ACE2* gene expression results, we performed a dot blot protein expression analysis. Briefly, the cancer cells were lysed following a 30-min incubation on ice with phosphate lysis buffer containing 0.1% imidazole, 0.5% triton X, and a 1X protease inhibitor and then vortexed. Cell lysates were spotted onto a nitrocellulose membrane and left to dry for 30 min at room temperature. The membrane was blocked with 3% bovine serum albumin (BSA) in 1X PBS-T and probed with primary antibodies. Unbound primary antibodies were washed out followed by secondary antibody incubation. The detection substrates were added and viewed using the AmershamTM Imager 600 (GE Life Sciences, UK). The data was quantified by measuring the density of each dot using the ImageJ version 1.53t (ImageJ software, National Institute of Health, USA). After background measurement, the readout was analyzed using the spreadsheet, and calculation was performed as detailed previously^45^.

### Cell proliferation assay

Roche cell proliferation kit 1 (MTT) (Sigma-Aldrich, St Louis, MO, USA; #11465007001) was used to determine cell proliferation post-infection following the manufacturer’s protocol. The cell lines were seeded into tissue-culture grade 96-well plates at a density of 1 × 10^4^ cells per well and 5 × 10^3^ cells per well for 22RV1 and DLD-1 respectively and incubated at 37 °C overnight. The cells were infected with sPV and incubated for 48 h and 72 h. MTT labeling reagent was added to each well and incubated at 37 °C for 4 h. Afterward, the solubilization buffer was added to each well and incubated at 37 °C overnight. Absorbance was read with Varioskan™ LUX microplate reader (Thermo Fisher Scientific, Carlsbad, CA, USA) at 570 nm to calculate percent cell growth. All incubations were done at 5% CO_2_.

### Cell viability assay

Cell viability was assessed using the Promega CellTiter-Glo® (#G9242) luminescent cell viability assay following the manufacturer’s instructions. Briefly, cells were seeded into tissue-culture grade 24-well plate at 1.5 × 10^5^ cells per well and incubated at 37 °C with 5% CO_2_ for not more than 24 h. The cells were then infected with sPV and incubated for 72 h with uninfected and mock viruses as controls. All assays were performed in triplicates. A volume of CellTiter-Glo® reagent equal to the volume of the cell culture medium present in each well was added and mixed gently on an orbital shaker for 2 min to lyse the cells. The plate was then incubated in the dark for 10 min at room temperature. The luminescence was read using the GloMax®-Multi detection system (Promega, USA).

### Wound healing assay

Wound-healing assay was performed with silicone culture inserts (Ibidi GmbH, Munich, Germany) as described previously^46^. Briefly, cells were seeded into a silicone culture insert placed in a 12-well plate at a density of 1 × 10^4^ cells per well and 5 × 10^3^ cells per well for 22RV1 and DLD-1 respectively and incubated at 37 °C with 5% CO_2_ overnight to allow the cells to attach to create about 90–95% confluent monolayer. The silicone culture inserts were removed gently and aseptically with sterile forceps. The plate was then washed gently thrice with sterile 1X PBS to remove non-attached cells. The cells were then infected and incubated at 37 °C and 5% CO_2_. Cell migration was monitored by taking images with an Optikalview light microscope (OPTIKA® Ponteranica, Italy) at 12-h time intervals. The images were analyzed with ImageJ software. The percentage (%) of the wound closure was calculated by using the percentage change in the normalized area measurement divided by the original open area according. That is, % = [Area (time zero)—Area (time after incubation)/Area (time zero)] × 100^47^.

### Data analysis

All the experiments were performed using at least three biological and technical replicates. Raw data was stored in Microsoft Excel sheets. Data normality was checked using the Shapiro–Wilk test. Statistical differences between two groups were performed using Wilcoxon rank-sum test. In case of three or more groups, *p*-vales were adjusted using rstatix package in R. Data were analyzed and visualized using freely available packages, particularly ggplot2 version 3.4.4, anchored in R version 4.3.2 (R Development Core Team, Vienna, Austria) and RStudio version 2023.09.1 + 494. All statistical tests are two-sided tests and statistical significance was considered at *p*-values less than 0.05.

## Supplementary Information

Supplementary Information.

## Data Availability

The data presented in this study are available in the article and its supplementary material.
