# Supplementary material for: In vitro analysis suggests that SARS-CoV-2 infection differentially modulates cancer-like phenotypes and cytokine expression in colorectal and prostate cancer cells
Source: Sci Rep. 2024 Oct 19;14:24625. doi: 10.1038/s41598-024-75718-1 (PMC11490510; doi:10.1038/s41598-024-75718-1)
Supplement: Supplementary file 1 — Supplementary Information. [file 41598_2024_75718_MOESM1_ESM.pdf]

***In vitro* analysis suggests that SARS-CoV-2 infection differentially modulates cancer-like phenotypes and cytokine expression in colorectal and prostate cancer cells.**

Alberta Serwaa[1,2], Fatima Oyawoye[1], Irene Amoakoh Owusu[1], Daniel Dosoo[1], Aaron Adom Manu[1,2], Augustine Kojo Sobo[1,2], Kwadwo Fosu[1,2], Charles Ochieng' Olwal[1,2], Peter Kojo Quashie[1,3]*, Anastasia Rosebud Aikins[1,2]*

[1] West African Centre for Cell Biology of Infectious Pathogens (WACCBIP), College of Basic and Applied Sciences, University of Ghana, Ghana
[2] Biochemistry, Cell, and Molecular Biology, College of Basic and Applied Sciences, University of Ghana, Accra, Ghana
[3] Francis Crick Institute, 1 Midland Road, London, NW1 1AT, United Kingdom

Figure S1. ACE2 expression in various cancer cell lines.

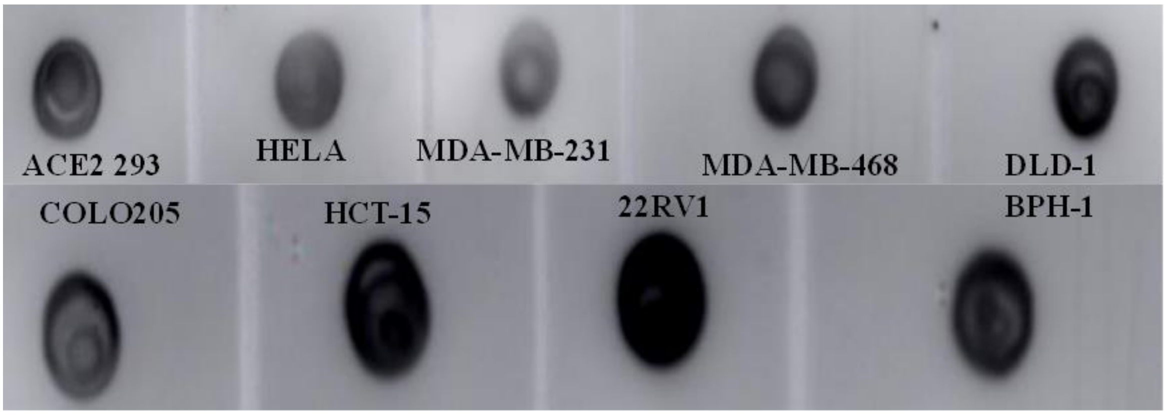

Figure S2. SARS-CoV-2 Infection may not significantly affect 22RV1 migration but reduces DLD-1 cell migration. A diagram showing representative wound healing images of (**a**) uninfected and SARS-CoV-2 infected 22RV1 cell lines at different time points and (**b**) uninfected and SARS-CoV-2 infected DLD-1 cell lines at different time points. Magnification x100. Clear dark area = wound area. White patched area = area covered with cells.

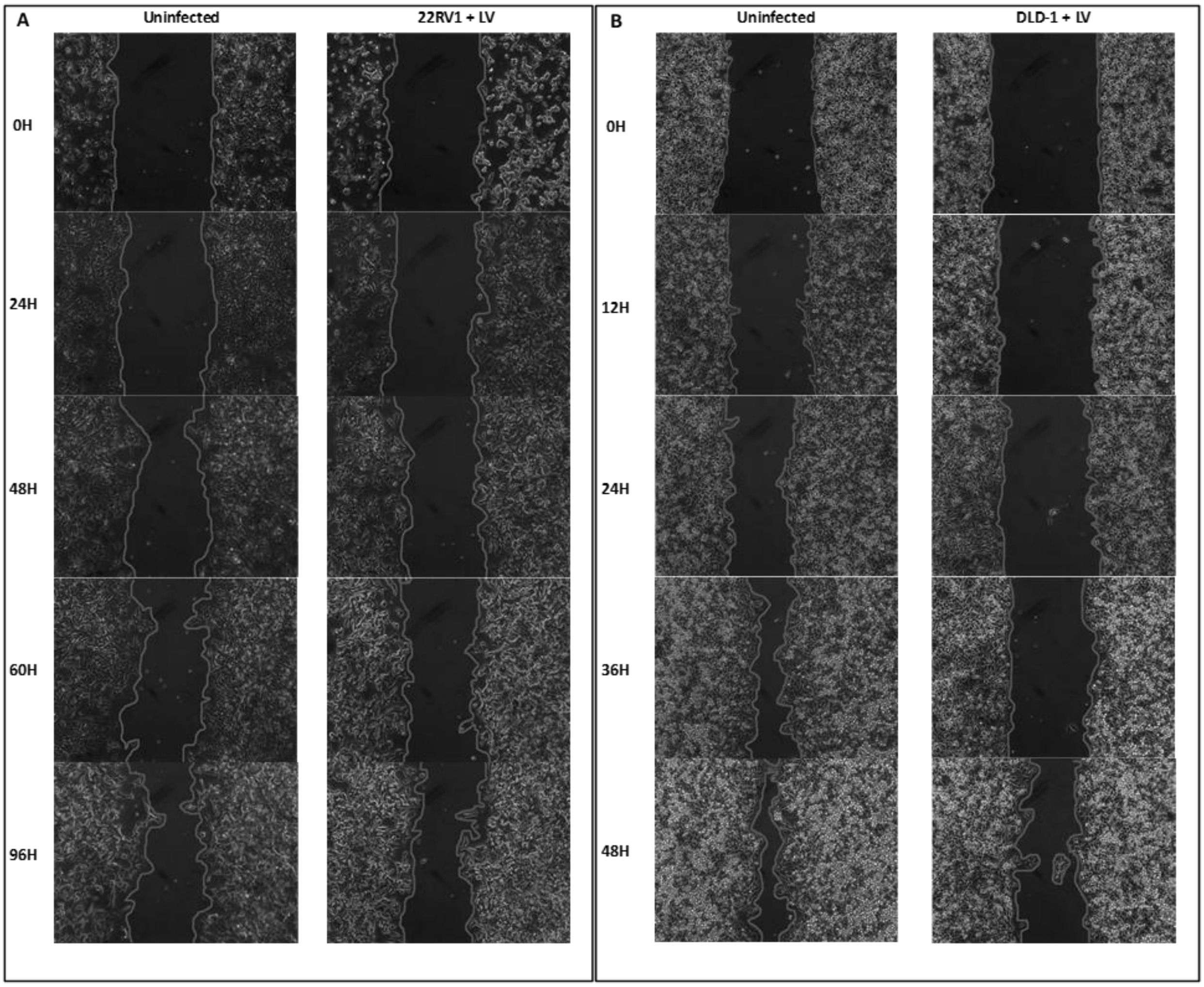

Tables

Table S1. Oligonucleotide sequences used in this study.

Target Gene	Set	Sequence (5'-3')	Annealing temperature (°C)	References
VEGF	Forward<br>Reverse	CTCTACCTCCACCATGCCAAGT<br>TCGATTGGATGGCAGTAGCTG	60	[1,2]
BCL-2	Forward<br>Reverse	TGCACCTGACGCCCTTCAC<br>AGACAGCCAGGAGAAATCAAACAG	59	[3]
KI-67	Forward<br>Reverse	AATTCAGACTCCATGTGCCTGAG<br>CTTGACACACACATTGTCCTCAGC	60	[4]
MMP9	Forward<br>Reverse	ACGCACGACGTCTTCCAGTA<br>CCACCTGGTTCAACTCACTCC	61	[5]
VIM	Forward<br>Reverse	TCTCTGAGGCTGCCAACCG<br>CGAAGGTGACGAGCCATTTCC	62	[6]
TNF-α	Forward<br>Reverse	AGTGACAAGCCTGTAGCCC<br>GCAATGATCCCAAAGTAGACC	57	[7]
IL-1β	Forward<br>Reverse	GCACGATGCACCTGTACGAT<br>CACCAAGCTTTTTTGCTGTGAGT	59	[8]
IL-6	Forward<br>Reverse	CAATGAGGAGACTTGCCTGGTGA<br>TGGCATTTGTGGTTGGGTCAG	62	[9,10]
IL-8	Forward<br>Reverse	CACCGGAAGGAACCATCTCACT<br>TCAGCCCTCTTCAAAAACTTCTCC	59	[10]
GAPDH	Forward<br>Reverse	TGCACCACCAACTGCTTA<br>GGATGCAGGGATGATGTTC	60	[11]

References

1. Herkenne, S. *et al.* Developmental and Tumor Angiogenesis Requires the Mitochondria-Shaping Protein Opa1. *Cell Metab.* **31**, 987-1003.e8 (2020).

2. Norol, F. *et al.* GFP-transduced CD34+ and Lin- CD34- hematopoietic stem cells did not adopt a cardiac phenotype in a nonhuman primate model of myocardial infarct. *Exp. Hematol.* **35**, 653–661 (2007).

3. Sun, H. *et al.* A newly identified G-quadruplex as a potential target regulating Bcl-2 expression. *Biochim. Biophys. Acta - Gen. Subj.* **1840**, 3052–3057 (2014).

4. Bai, Y. *et al.* Ki-67 is overexpressed in human laryngeal carcinoma and contributes to the proliferation of HEp2 cells. *Oncol. Lett.* **12**, 2641–2647 (2016).

5. Fan, Y. *et al.* NLRC5 promotes cell migration and invasion by activating the PI3K/AKT signaling pathway in endometrial cancer. *J. Int. Med. Res.* **48**, 1–13 (2020).

6. Sittiju, P. *et al.* Osteosarcoma-Specific Genes as a Diagnostic Tool and Clinical Predictor of Tumor Progression. *Biology (Basel).* **11**, 1–14 (2022).

7. Zhao, C., Lu, X., Bu, X., Zhang, N. & Wang, W. Involvement of tumor necrosis factor-α in the upregulation of CXCR4 expression in gastric cancer induced by Helicobacter pylori. *BMC Cancer* **10**, 419 (2010).

8. Bhat, I. A. *et al.* Association of interleukin 1 beta (IL-1β) polymorphism with mRNA expression and risk of non small cell lung cancer. *Meta Gene* **2**, 123–133 (2014).

9. Liu, D., Perkins, J. T., Petriello, M. C. & Hennig, B. Exposure to coplanar PCBs induces endothelial cell inflammation through epigenetic regulation of NF-κB subunit p65. *Toxicol. Appl. Pharmacol.* **289**, 457–465 (2015).

10. Zhao, W. *et al.* Activation of Rev-erbα attenuates lipopolysaccharide-induced inflammatory reactions in human endometrial stroma cells via suppressing TLR4-regulated NF-κB activation. *Acta Biochim. Biophys. Sin. (Shanghai).* **51**, 908–914 (2019).

11. Wu, D. *et al.* Long noncoding RNA NNT-AS1 enhances the malignant phenotype of bladder cancer by acting as a competing endogenous RNA on microRNA-496 thereby increasing HMGB1 expression. *Aging (Albany. NY).* **11**, 12624–12640 (2019).
